# 

**Published:** 1902-10-25

**Authors:** 


					tee hospital. " The standard of highest purity."?Lancet. Oct. 25th, 1902.
CADBURY'S w* to. CADBURY'S
cocoa rl aPm Im oocoa
ABSOLUTELY PURE. Jft, DRUGS OR ALKALIES UIBD.
Ko. 839. Vol. XXXIII. |JSSSJ?] SATURDAY,
Oct. 25th, 1902.
[Sabscnptionjerma,] pRICH TWOPENCE.]

				

